# Postprandial Increases in Liver-Gut Hormone LEAP2 Correlate with Attenuated Eating Behavior in Adults Without Obesity

**DOI:** 10.1210/jendso/bvad061

**Published:** 2023-05-12

**Authors:** Raghav Bhargava, Sandra Luur, Marcela Rodriguez Flores, Mimoza Emini, Christina G Prechtl, Anthony P Goldstone

**Affiliations:** PsychoNeuroEndocrinology Research Group, Division of Psychiatry, Department of Brain Sciences, Faculty of Medicine, Imperial College London, Hammersmith Hospital, London, W12 0NN, UK; PsychoNeuroEndocrinology Research Group, Division of Psychiatry, Department of Brain Sciences, Faculty of Medicine, Imperial College London, Hammersmith Hospital, London, W12 0NN, UK; PsychoNeuroEndocrinology Research Group, Division of Psychiatry, Department of Brain Sciences, Faculty of Medicine, Imperial College London, Hammersmith Hospital, London, W12 0NN, UK; PsychoNeuroEndocrinology Research Group, Division of Psychiatry, Department of Brain Sciences, Faculty of Medicine, Imperial College London, Hammersmith Hospital, London, W12 0NN, UK; School of Public Health, Faculty of Medicine, Imperial College London, St. Mary's Hospital, London, W2 1PG, UK; PsychoNeuroEndocrinology Research Group, Division of Psychiatry, Department of Brain Sciences, Faculty of Medicine, Imperial College London, Hammersmith Hospital, London, W12 0NN, UK

**Keywords:** ghrelin, LEAP2, fMRI, food cue reactivity, appetite, insulin, glucose, triglycerides

## Abstract

**Background:**

The novel liver-gut hormone liver-expressed antimicrobial peptide-2 (LEAP2) is a centrally acting inverse agonist, and competitive antagonist of orexigenic acyl ghrelin (AG), at the GH secretagogue receptor, reducing food intake in rodents. In humans, the effects of LEAP2 on eating behavior and mechanisms behind the postprandial increase in LEAP2 are unclear, though this is reciprocal to the postprandial decrease in plasma AG.

**Methods:**

Plasma LEAP2 was measured in a secondary analysis of a previous study. Twenty-two adults without obesity attended after an overnight fast, consuming a 730-kcal meal without or with subcutaneous AG administration. Postprandial changes in plasma LEAP2 were correlated with postprandial changes in appetite, high-energy (HE) or low-energy (LE) food cue reactivity using functional magnetic resonance imaging, *ad libitum* food intake, and plasma/serum AG, glucose, insulin, and triglycerides.

**Results:**

Postprandial plasma LEAP2 increased by 24.5% to 52.2% at 70 to 150 minutes, but was unchanged by exogenous AG administration. Postprandial increases in LEAP2 correlated positively with postprandial decreases in appetite, and cue reactivity to HE/LE and HE food in anteroposterior cingulate cortex, paracingulate cortex, frontal pole, and middle frontal gyrus, with similar trend for food intake. Postprandial increases in LEAP2 correlated negatively with body mass index, but did not correlate positively with increases in glucose, insulin, or triglycerides, nor decreases in AG.

**Conclusions:**

These correlational findings are consistent with a role for postprandial increases in plasma LEAP2 in suppressing human eating behavior in adults without obesity. Postprandial increases in plasma LEAP2 are unrelated to changes in plasma AG and the mediator(s) remain uncertain.

Acyl ghrelin (AG) is a 28-amino acid orexigenic hormone principally secreted by the oxyntic cells of the fundus and body of the stomach that stimulates preparation for feeding and food intake via actions as an agonist at the GH secretagogue receptor 1a (GHSR1a) in a number of brain regions, including the hypothalamus, midbrain, and hippocampus, as well as indirectly via the vagus nerve [1, 2]. Ghrelin also exists in an inactive form des-acyl ghrelin (DAG), which is not a GHSR1a agonist, and is acylated to AG by the ghrelin-O-acyl transferase (GOAT) enzyme [3].

Liver-expressed antimicrobial peptide-2 (LEAP2) is a recently discovered circulating anorexigenic hormone [4], originally isolated from human monocytes [5], that acts as a competitive antagonist of orexigenic AG [6] and inverse agonist [4, 7] at the constitutively active GHSR1a. Mature LEAP2 is a 40-amino acid peptide [5, 8], with identical structure in rodents and humans. It is expressed principally in the liver and small intestine [5, 9]. In rodents, peripheral and/or central administration of LEAP2 blocks the orexigenic actions of peripheral and/or central administration of AG [4, 9‐11], and LEAP2 may reduce food intake when administered alone [4, 11‐13].

Study of *Ghrl*, *Ghsr1a*, and *Goat* knockout mice and use of GHSR1a antagonists or inverse agonists in rodents supports a direct role for endogenous AG and GHSR1a signaling to stimulate food intake and impulsivity in animals, especially under conditions of stress and after food restriction [2, 14‐20]. However, there is limited direct evidence for a role of endogenous plasma AG in human eating behavior, including in the fed state, as increases in appetite and food intake are not seen after physiological, as opposed to pharmacological, exogenous AG administration [21‐24]. Correlations of the postprandial decreases in endogenous plasma AG with postprandial decreases in appetite and food intake are infrequently seen (see Supplementary References for a list of publications [25]).

In the only human study to date, exogenous full-length LEAP2 infusion in men without obesity, when fed to produce mildly supraphysiological plasma LEAP2 concentrations, acutely reduced food intake but not appetite ratings, supporting a stimulatory role of GHSR1a signaling on human food intake [13].

In rodents and humans, both plasma AG and LEAP2 have a reciprocal relationship with nutritional status, with plasma AG decreasing, and in most studies plasma LEAP2 increasing, after food intake, with reciprocal responses in the acute fasted state [4, 9, 13, 26‐28]. Plasma LEAP2 is increased, whereas plasma AG is decreased, in obesity [27, 29‐31]. Plasma AG increases with dietary-induced weight loss [32, 33], whereas LEAP2 falls after weight loss by both dietary restriction in rodents and bariatric surgery in humans [4, 27, 31, 34]. Therefore, changes in the plasma AG/LEAP2 ratio by nutritional status may be an important driver to regulate appetite and food intake during energy homeostasis [27].

Postprandial decreases in plasma ghrelin may be via increases in circulating insulin or changes in autonomic nervous system innervation of stomach [2, 35‐46]. However, any role for circulating insulin in increasing plasma LEAP2 is currently unknown.

Furthermore, it is incompletely understood how and from where plasma LEAP2 increases after food intake. In rodents, *Leap2* mRNA expression decreased with fasting and increased after refeeding only from the liver but not small intestine [9, 31], though this has yet to be examined in humans. In mice without obesity and humans with obesity, plasma glucose positively correlated with plasma LEAP2 across fasting and postprandial time points, suggesting that glucose might stimulate LEAP2 secretion after meals in humans [27]. Postprandial increases in plasma LEAP2 were also positively correlated with body mass index (BMI) across a combined group of adults with normal weight and obesity [27]. Another possible mediator of the postprandial increase in plasma LEAP2 might be the reciprocal postprandial decrease in plasma AG, as well as circulating, direct intraluminal intestinal, or indirect hepatic portal vein effects of glucose or other nutrients.

The sites of action of LEAP2 in the human brain remain to be determined, but animal studies have implicated actions on hypothalamic orexigenic NPY and anorexigenic POMC neurons [9, 27, 34, 47, 48].

In humans, functional magnetic resonance imaging (fMRI) has been increasingly used to understand how food-related tasks, such as looking at or rating food pictures or words (food cue reactivity), activate specific areas of the brain, especially those involved in reward processing and decision making [49, 50]. Nutritional state influences food cue reactivity with food intake decreasing food appeal and blood oxygen level-dependent (BOLD) signal to food pictures in several brain regions, including those involved in reward processing, emotional responses, and decision making, which may depend on the nature, salience, palatability, and energy density of the food cues, as well as obesity status [51‐57].

The peripheral signals that mediate these effects of nutritional state on food cue reactivity likely include circulating appetitive hormones that act on the brain [58], such as the decrease in plasma AG with food intake and increase in plasma AG with acute fasting [2], and potentially the reciprocal changes in plasma LEAP2.

However, results from fMRI studies investigating correlations of the postprandial decrease in endogenous plasma ghrelin with food cue reactivity in brain regions involved in reward, motivation and emotion have been inconsistent, likely related to often small samples sizes, differences in study design and obesity status, and specificity of ghrelin immunoassays to AG, DAG, or both [56, 59‐68).

To our knowledge, there have been no published studies of the effects of exogenous LEAP2 administration on or correlations of plasma LEAP2 with food cue reactivity using fMRI or measures of food hedonics. We therefore hypothesized that as AG stimulates appetite and food intake, cue reactivity and hedonics, the opposite would be seen for LEAP2, and so postprandial increases in plasma LEAP2, and/or postprandial decreases in plasma AG or AG/LEAP2 ratio, would be: (1) positively correlated with postprandial decreases in appetite ratings; (2) positively correlated with food cue reactivity (especially to high-energy [HE] foods); and/or (3) positively correlated with food intake, whereas (4) the postprandial changes in these appetitive hormones would be positively correlated with postprandial increases in potential metabolic or hormonal mediators, and (5) if AG directly suppresses LEAP2 secretion, exogenous AG administration would lower plasma LEAP2 when fed and/or the postprandial increase in plasma LEAP2 would be positively correlated with the postprandial decrease in plasma AG.

Plasma LEAP2 was therefore measured in a secondary analysis of our previous study comparing effects of exogenous and endogenous hyperghrelinemia on eating behavior [56], in which adults without obesity had visits after an overnight fast, or after consuming a breakfast meal without or with prior subcutaneous AG administration, with measurement of appetite ratings, food cue reactivity using a food picture evaluation fMRI task measuring BOLD signal from whole brain analysis, food intake at an *ad libitum* lunch meal, and plasma glucose, serum insulin, and triglycerides.

## Material and Methods

Healthy adults without obesity of either sex aged 18 to 50 years participated in a randomized, placebo-controlled crossover design study with 3 visits at least 6 days apart: (1) after a 16-hour overnight fast (fasted-saline) or after consuming mixed breakfast (at t = 0 minutes at ∼10:00) 95 minutes before scanning (730 kcal, 14% protein, 31% fat, 55% carbohydrate) and 40 minutes before receiving a subcutaneous (2) saline injection (fed-saline) or (3) AG injection at 3.6 nmol/kg (fed-ghrelin) before scanning, followed by an *ad libitum* lunch meal 150 minutes after breakfast, as previously described [56] (Supplementary Fig. S1 [25]). Participants were in the MRI scanner between t = +75 and +135 minutes, with the food picture evaluation fMRI task between t = +95 and +115 minutes. Further details of the study design are available in the earlier publication [56]. All women attended in the first 14 days of their menstrual cycle.

The study was approved by the local Hammersmith and Queen Charlotte's & Chelsea Research Ethics Committee (07/Q0406/19) and was performed in accordance with the principles of the Declaration of Helsinki. All participants provided written informed consent.

### Blood Sampling and Hormone Assays

At each visit, blood samples were taken at t = −15, 0, +40, +70, and +150 minutes. Plasma glucose, serum insulin, and triglycerides were determined with routine laboratory techniques as reported previously [56]. Homeostasis model assessment of insulin resistance was calculated using the formula: fasting insulin (mU/L) × fasting glucose (mmol/L)/22.5 [69], averaging the results from the t = −15 and 0 minutes time points at fasted-saline and fed-saline visits. Plasma samples were stored at −80 °C for future measurement of hormones. Plasma AG was measured at t = −15, 0, +40, +70, and +150 minutes from lithium heparin samples containing AEBSF protease inhibitor (4-(2-aminoethyl)benzenesulfonyl fluoride hydrochloride) (A8456; Sigma-Aldrich) with acidification of plasma using hydrochloric acid, with a previously validated ELISA, University of Virginia [26, 70], as reported previously [56].

For this study, plasma LEAP2 concentrations were measured at time points t = −15, 0, +70, and +150 minutes using a commercial enzyme immunoassay (EK-075-40, Phoenix Pharmaceuticals Inc., CA, USA) according to the manufacturer's instructions using lithium heparin samples containing AEBSF protease inhibitor (without acidification of plasma). This LEAP2 assay has been previously validated using human samples [27]. Plasma samples were diluted 10 times in the assay buffer. The inter-assay and intra-assay coefficients of variation were 12.5% and 8.2%, respectively, both within the manufacturer guidance of <15% and <10%.

The plasma AG/LEAP2 molar ratio was calculated by dividing the results for each assay after converting concentrations to pmol/L. For plasma LEAP2, AG, and AG/LEAP2 molar ratio, within-participant postprandial changes were calculated by subtracting the value for the fasted-saline from the fed-saline visit. Because of the timing of individual outcome measures over the study visit, the timing of hormone values used for correlations with eating behavior were as follows: appetite and food cue reactivity, average of t = +70 and +150 minutes time points (pre-MRI, and post-MRI/pre-lunch, respectively); and food intake at *ad libitum* lunch, t = +150 minutes (post-MRI/pre-lunch). For correlations within appetitive hormones, and between appetitive hormones and plasma glucose, serum insulin, and triglycerides, the difference in the incremental area under curve (iAUC) between 0 and 150 minutes (iAUC_0-150 minutes_) was calculated between fed-saline and fasted-saline visits.

### Appetite Visual Analog Scale Ratings

Serial visual analog scale (VAS) ratings (0-10 cm) were used to assess hunger, pleasantness to eat, prospective volume able to eat, and fullness, as previously reported [56]. For data reduction and to reduce issues with multiple comparisons, these VAS results were used to calculate a composite appetite VAS rating as: appetite = [hunger + pleasantness + volume + (10 – fullness)]/4 for the 2 time points (t = +70 and +150 minutes). Within-participant postprandial changes of composite appetite and fullness VAS ratings were calculated using the average of t = +70 and +150 minutes at each visit and then subtracting the value for fasted-saline from the fed-saline visit.

### Food Picture Evaluation fMRI Task

As previously described, during the fMRI task, participants were requested to rate the picture appeal while simultaneously viewing pictures of HE foods, LE foods, household objects, and blurred pictures [56]. The total fMRI task lasted 20 minutes using a block design in 2 runs. Within each run, pictures were presented in a randomized order with a pseudorandomized order of 5 blocks of HE-density foods, 5 blocks of LE-density foods, and 5 blocks of objects, with 16 blocks of blurred images shown between the other blocks. Picture appeal was rated simultaneously using a keypad with 5 buttons. Ratings ranged from 1 to 5, with 1 = not at all, 2 = not really, 3 = neutral, 4 = a little, and 5 = a lot on the scale of picture appeal [56]. Within-participant postprandial changes of picture appeal for HE food vs. objects and LE food vs objects was calculated as the difference between fed-saline and fasted-saline visits for this analysis.

### MRI Scanning Protocol and fMRI preprocessing

This is given in detail in the original publication [56], which included motion correction, field map-based unwarping, spatial smoothing, high-pass temporal filtering, and inclusion of temporal derivative and motion variables as covariates [56]. For this analysis, the scans were reprocessed to now include boundary-based registration to improve spatial registration between the low-resolution echoplanar fMRI images and high-resolution T1 anatomical scans in subject space.

### Whole Brain Analysis

The fMRI data processing was carried out using FEAT (FMRI Expert Analysis Tool) Version 6.00, part of FSL (FMRIB's Software Library, www.fmrib.ox.ac.uk/fsl). Whole-brain analysis was used to first combine the 2 runs of the fMRI task and then to determine differences in BOLD signal between fed-saline and fasted-saline visits using a paired *t*-test with fixed effects analyses for each participant. This was done separately for HE food vs. object, LE food vs. object, HE/LE food vs. object, and HE vs. LE food contrasts.

For whole-brain analysis, to examine correlations between the postprandial changes in BOLD signal for each contrast with postprandial changes in each plasma hormone (LEAP2, AG, AG/LEAP2 molar ratio), the demeaned hormone value for fed-saline minus fasted-saline visits for the average of t = +70 and +150 minute time points were entered into the general linear model as an explanatory variable using FEAT, using a mixed effects analysis with threshold cluster-wise family-wise error *Z* >2.3, *P* < .05, to correct for multiple comparisons.

### Energy Intake

During the fasted-saline visit, participants did not receive breakfast at t = +0 minutes. At the fed-saline and fed-ghrelin visits, a 730-kcal standardized fixed breakfast was given to the participants.

After the fMRI session at t = +150 minutes, participants were served an excess amount of savory lunch, consisting of macaroni and cheese (per 100 g: 205 kcal, 6.5 g protein, 19.9 g carbohydrate, 11.5 g fat) or (if the former was not liked having been tested at the screening visit, n = 5) chicken tikka masala (per 100 g: 150 kcal, 6.6 g protein, 13 g carbohydrate, 8 g fat). Participants were told to consume the lunch until they felt adequately full. The total amount of food presented varied between sexes: men were given 2000 g and women 1500 g.

At each visit, participants had their body fat percentage determined by bioelectrical impedance analysis (Bodystat 1500, Bodystat Ltd., UK). Estimated resting energy expenditure (REE) in kcal was calculated for each participant: (1) incorporating fat free mass (kg) from bioimpedance analysis (BIA) using the Cunningham equation: (22 × free fat mass) + 500 (equating lean body mass with fat-free mass) [71]; and (2) incorporating weight (kg) adjusting for age and sex using the Schofield equation—males (ages 18-29 years): (15.057 × weight) + 692.2; males (ages 30-59 years): (11.472 × weight) + 873.1; females (ages 18-29 years): (14.818 × weight) + 486.6; females (ages 30-59 years): (8.126 × weight) + 845.6 [72].

Total energy intake was expressed as total kcal consumed expressed as a percentage of 24-hour estimated REE using the Cunningham equation as primary outcome. Correlations of plasma hormones with total energy intake also used REE-adjusted values from Schofield equation to avoid any potential confounds from BIA % body fat measurements, for example variability in hydration status (though participants had free access to water throughout the study visits). The original publication demonstrated no significant difference in BMI or % body fat between fed-saline and fasted-saline visits. Percentage body fat was also compared between these visits using intraclass correlation coefficients to further exclude any factors that may have influenced the calculation of fat-free mass using BIA between visits.

Reported BMI and % body fat were calculated from measurements of height and weight and averaged from fed-saline and fasted-saline visits.

### Statistical Analyses

Data are presented as mean ± standard error of the mean (SEM) or median (interquartile range [IQR]), where it was not normally distributed. Data were checked for normal distribution using the Shapiro-Wilk test.

Comparisons between visits (fed-saline vs. fasted-saline, or fed-saline vs. fed-ghrelin) using multiple time points for appetitive hormones was made using a 2-way repeated measures ANOVA including visit and time as within-participant factors, with post hoc Tukey test. Between visit comparison for single measurements (iAUC_0-150minutes_ for hormones, appetite/fullness VAS ratings, food intake [as % REE]) were made using paired Student *t*-test for normally distributed data or Wilcoxon matched-pairs signed-rank test where data were not normally distributed. Comparisons between visits (fed-saline vs. fasted-saline) for food appeal rating was made using a 2-way repeated measures ANOVA including visit and energy density (HE food, LE food) as within-participant factors, with post hoc Tukey test. Effect sizes were reported from these ANOVA and *t*-test analyses.

Correlation analysis was performed to examine the relationships between outcome variables (without any covariates) reporting Pearson (r_P_) if data were normally distributed, or Spearman (r_S_) correlation coefficients if ether variable was not normally distributed. This was used to correlate relationships between plasma AG, LEAP2, and AG/LEAP2 molar ratio (dependent variable) and appetite/fullness VAS ratings, food intake (as % REE), plasma glucose, serum insulin and triglycerides concentrations, homeostasis model assessment of insulin resistance, and BMI values (independent variable), and also between plasma LEAP2 and AG. Statistical significance was set to *P* < .05. Statistical analysis used IBM SPSS v27 (Armonk, NY, USA) and GraphPad Prism v9.5.0 (San Diego, CA, USA).

## Results

### Participant Characteristics

As previously reported [56], 22 adults without obesity, predominately men, completed all 3 study visits, with time between visits median (IQR) 16 (7, 35) days (Table 1). Intraclass correlation coefficient for % body fat using BIA between fasted-saline and fed-saline visits was 0.98, *P* < .001.

**Table 1.** Participant characteristics

	Mean ± SD	Range
N	22	
Age (y)	25.9 ± 8.0	19-44
Male, n (%)	17 (72.3%)	
Ethnicity White, n (%)	15 (68.1%)	
Height (m)	1.78 ± 0.09	1.61-1.93
Weight (kg)	76.0 ± 13.6	51.6-100.9
BMI (kg/m^2^)	23.9 ± 2.8	19.1-29.9
Body fat (%)	16.1 ± 6.6	7.8-30.7

Abbreviation: BMI, body mass index.

### Postprandial Increase in Plasma LEAP2

**Figure 1.**
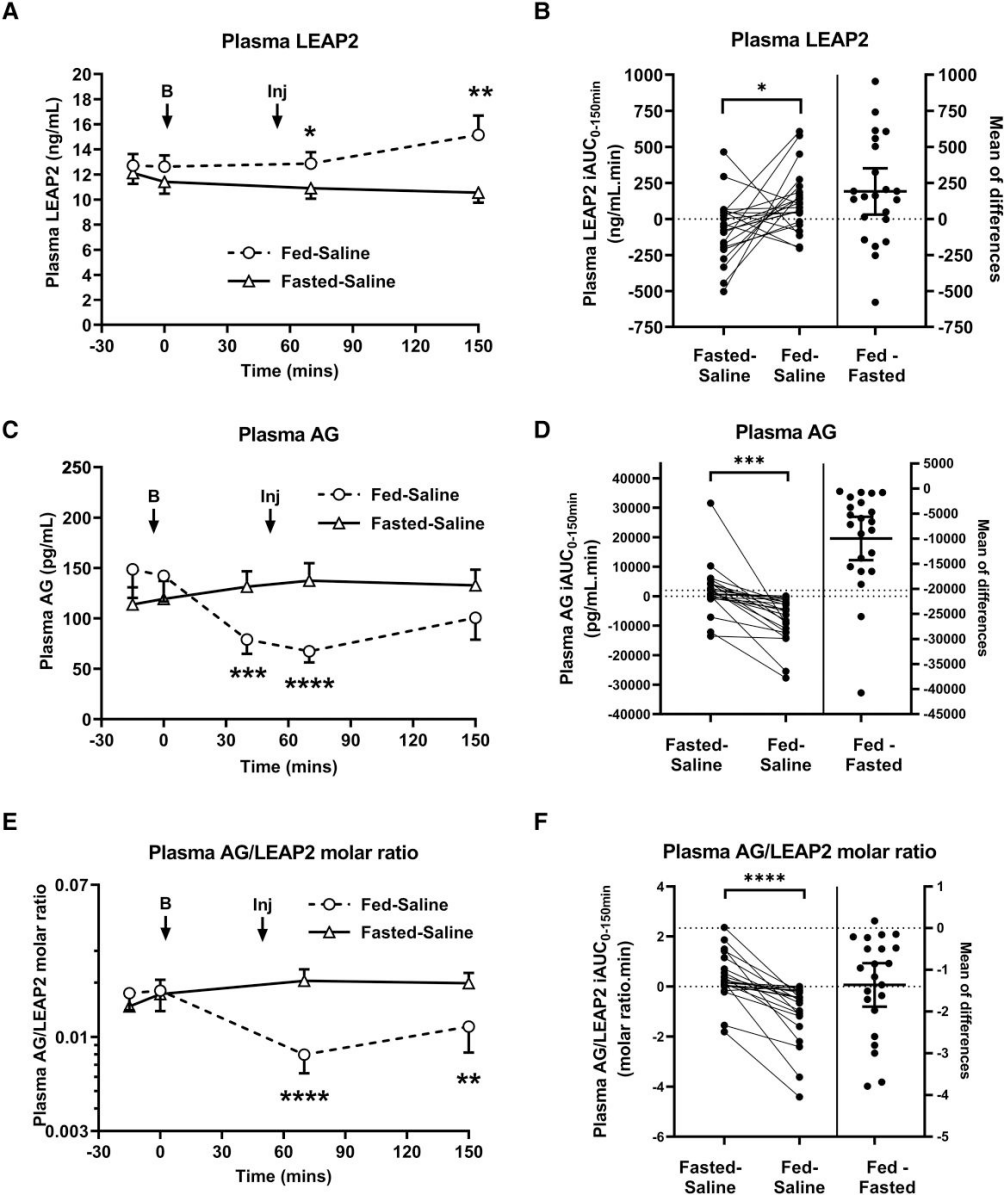
**Plasma LEAP2, AG, and AG/LEAP2 molar ratio concentrations at study visits.** (A, C, E) Plasma hormone concentrations over study visits for (A) LEAP2, (C) AG, and (E) AG/LEAP2 molar ratio at fasted-saline and fed-saline visits. Arrows indicate (B) time of breakfast (t = 0 minutes) and (Inj) subcutaneous saline/AG injection (t = +55 minutes). Comparisons made using 2-way repeated measures ANOVA with post hoc Tukey test: *P < .05, **P < .01, ***P < .001, ****P < .0001 fed-saline vs. fasted-saline, n = 22. (B, D, F) Comparison of incremental value of area under the curve (iAUC) from t = 0 to +150 minutes for plasma (B) LEAP2, (D) AG, and (F) AG/LEAP2 molar ratio between fed-saline and fasted-saline visits with right-hand part of graph showing mean differences between visits (Δ fed-fasted). Comparisons made using (B) paired Student *t* test and (D, F) Wilcoxon matched-pairs signed-rank test: *P < .05, **P < .01, ***P < .001, ****P < .0001, n = 22. All data given as mean ± SEM. Abbreviations: AG, acyl ghrelin.

In repeated measures ANOVA, including visit (fed-saline, fasted-saline) and time (t = 0, 70, 150 minutes) as within-participant factors, there was a significant time × visit interaction for plasma LEAP2 [F(3,63) = 6.05, *P* = .001]. Plasma LEAP2 increased after food intake at both t = 70 and 150 minute time points (Fig. 1A).

In post hoc analysis, at t = 70 minutes after food intake, plasma LEAP2 was mean ± SEM 24.5 ± 7.8% higher (*P* < .001) at the fed-saline (mean ± SEM 12.87 ± 0.91 ng/mL) than the fasted-saline (10.90 ± 0.84 ng/mL) visit (effect size mean ± SEM, 1.96 ± 0.63 ng/mL [95% CI 0.38, 3.55], *P* = .014]. At t = 150 minutes after food intake, plasma LEAP2 was 52.2 ± 13.8% higher (*P* = .002) at the fed-saline visit (15.15 ± 1.55 ng/mL) than the fasted-saline visit (10.56 ± 0.80 ng/mL) (effect size 4.59 ± 1.31 ng/mL [95% CI 1.29, 7.90], *P* = .006).

Plasma LEAP2 iAUC_0-150 minutes_ was significantly higher at the fed-saline (119.37 ± 45.68 ng/mL.min) than fasted-saline (−72.23 ± 46.65 ng/mL.min) visit (effect size 191.61 ± 77.13 ng/mL.min [95% CI 28.35, 340.10], t = 2.48, *P* = .021, paired Student *t* test) (Fig. 1B).

When comparing the absolute postprandial increase in plasma LEAP2 across this study [56], and the four analyses from three previously published studies [27, 28, 31] (overall two without obesity, three with obesity), there was a suggestion that the postprandial increase in plasma LEAP2 was greater with increasing energy intake at the meal, although with the small sample size this did not reach statistical significance (r_s_ = + 0.70, *P* = .23) (Supplementary Fig. S2 [25]).

### Postprandial Increase in LEAP2 is Unaffected by Exogenous AG Administration

In repeated measures ANOVA, including visit (fed-saline, fed-ghrelin) and time (t = 0, 70, 150 minutes) as within-participant factors, there was no significant time × visit interaction [F(3,63) = 0.323, *P* = .81] on plasma LEAP2 (Supplementary Fig. S3A [25]). There was also no significant overall effect of visit (independent of time) [F(1,21) = 0.09, *P* = .77], but there was an overall significant effect of time (independent of visit) [F(3,63) = 11.54, *P* <.0001].

Plasma LEAP2 iAUC_0-150 minutes_ was similar at the fed-saline (median 92.38 ng/mL.min [IQR −23.2, 196.8]) and fed-ghrelin (median 101.2 ng/mL.min [IQR −21.3, 297.9]) visit (W-statistic 55, *P* = .38, Wilcoxon matched-pairs signed rank test) (Supplementary Fig. S3B (25)).

At t = 70 minutes, plasma LEAP2 was significantly higher by 25.7 ± 7.6% (*P* < .001) when averaged across both fed visits (12.82 ± 0.75 ng/mL) compared with the fasted-saline visit (10.90 ± 0.84 ng/mL) (effect size 1.92 ± 0.63 ng/mL [95% CI 0.61, 3.22], t = 3.05, *P* = .006, paired Student *t* test). At t = 150 minutes, plasma LEAP2 was higher by 51.6 ± 11.4% (*P* < .001) when averaged across both fed visits (14.98 ± 1.10 ng/mL) compared with the fasted-saline visit (10.56 ± 0.80 ng/mL) (effect size 4.42 ± 0.88 ng/mL [95% CI 2.58, 6.26], t = 5.00, *P* < .0001, paired Student *t* test).

Averaged across both fed visits, plasma LEAP2 was significantly higher by 22.5 ± 4.8% (*P* < .001) at t = 150 minutes (14.98 ± 1.10 ng/mL), than at t = 0 minutes (12.23 ± 0.76 ng/mL) (effect size 2.75 ± 0.50 [95% CI 1.42, 4.08], t = 4.76, *P* < .0001, paired Student *t* test). However, plasma LEAP2 at t = 70 minutes (12.82 ± 0.75 ng/mL) was not significantly different (higher by 5.68 ± 3.17%, *P* = .15) from t = 0 minutes (effect size 0.60 ± 0.50 [95% CI −0.73, 1.92], t = 1.74, *P* = .64, paired Student *t* test).

### Postprandial Decrease in Plasma AG

As previously reported [56], in repeated measures ANOVA, including visit (fed-saline, fasted-saline) and time (t = 0, 40, 70, 150 minutes) as within-participant factors, there was a significant time × visit interaction on plasma AG [F(4,84) = 13.17, *P* < .0001].

In post hoc analysis, plasma AG decreased after food intake at both t = 70 and 150 minute time points (Fig. 1C). At t = 70 minutes, plasma AG was significantly lower by 47.3 ± 4.3% (*P* < .001) at the fed-saline (67.36 ± 11.03 pg/mL) than the fasted-saline (137.38 ± 17.44 pg/mL) visit (effect size −70.02 ± 12.73 pg/mL [95% CI −94.97, −45.06], t = −5.5, *P* < .0001]. However, at t = 150 minutes, plasma AG was lower by 23.4 ± 8.9% (*P* = .08) at the fed-saline (100.63 ± 21.70 pg/mL) than the fasted-saline (132.76 ± 15.51 pg/mL) visit (effect size −32.14 ± 18.06 pg/mL [95% CI −69.69, 5.42], t = −2.54, *P* = .089).

Plasma AG iAUC_0-150 minutes_ was significantly lower at the fed-saline (median −5713 pg/mL.min, [IQR −12,194, −2,125]) than fasted-saline (median 1,391 pg/mL.min, [IQR −524, 4,254]) visit (W-statistic −253, *P* < .0001, Wilcoxon matched-pairs signed rank test) (Fig. 1D).

### Postprandial Decrease in Plasma AG/LEAP2 Molar Ratio

In repeated measures ANOVA, including visit (fed-saline, fasted-saline) and time (t = 0, 70, 150 minutes) as within-participant factors, there was a significant time × visit interaction on plasma AG/LEAP2 molar ratio [F(2,42) = 15.56, *P* < .0001].

In post hoc analysis, plasma AG/LEAP2 molar ratio decreased after food intake at both t = 70 and 150 minute time points (Fig. 1E). At t = 70 minutes, plasma AG/LEAP2 molar ratio was 53.7 ± 5.2% lower (*P* < .001) at the fed-saline (0.0079 ± 0.0016) than the fasted-saline (0.0205 ± 0.0032) visit (effect size −0.0125 ± 0.0017 [95% CI −0.0168, −0.0082], *P* < .0001). At t = 150 minutes, plasma AG/LEAP2 molar ratio was 43.9 ± 7.2% lower (*P* < .001) at the fed-saline (0.0114 ± 0.0032) than the fasted-saline (0.0198 ± 0.0028) visit (effect size −0.0084 ± 0.0017 [95% CI −0.0127, −0.0042], *P* < .0001).

Plasma AG/LEAP2 molar ratio iAUC_0-150 minutes_ was significantly lower at the fed-saline (median −0.51 molar ratio.min [IQR −1.29, −0.20]) than fasted-saline (median 0.17 molar ratio.min, [IQR 0.04, 0.81]) visit (W-statistic −247, *P* < .0001, Wilcoxon matched-pairs signed-rank test) (Fig. 1F).

### Correlations Between Change in Postprandial Plasma LEAP2 and AG Concentrations

The postprandial increase in plasma LEAP2 iAUC_0-150 minutes_ in fact showed a trend to negatively correlate with the postprandial decrease in plasma AG iAUC_0-150 minutes_ (r_s_ = −0.37, *P* = .086) (Supplementary Fig. S3C [25]).

### Postprandial Decreases in Appetite and Increases in Fullness

**Figure 2.**
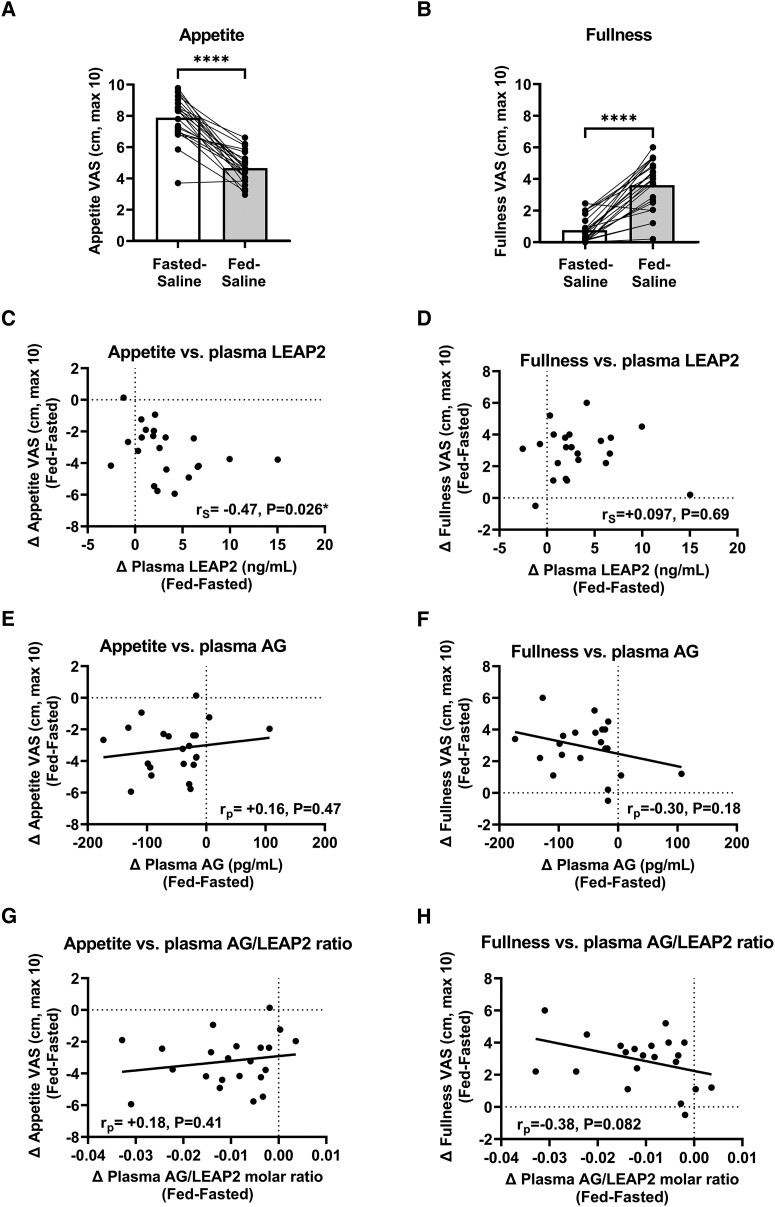
**Appetite and fullness ratings at fed and fasted visits and correlations of postprandial plasma LEAP2, AG, and AG/LEAP2 molar ratio with appetite and fullness ratings.** Visual analog scale (VAS) ratings of (A) appetite and (B) fullness (average of t = +70 and +150 minutes) between fed-saline (730 kcal breakfast) and fasted-saline (overnight fast) visits with comparisons made using paired Student *t* test: *P < .05, **P < .01, ***P < .001, ****P < .0001, n = 22. (C-H) Correlation between changes in (C, E, G) appetite and (D, F, H) fullness VAS ratings and changes in hormones (average of t = +70 and +150 minutes) between fed-saline and fasted-saline visits (Δ fed-fasted) for plasma (C, D) LEAP2, (E, F) AG, and (G, H) AG/LEAP2 molar ratio using Pearson (r_P_, linear regression line) or Spearman (r_S_) correlation coefficients, n = 22. Abbreviations: AG, acyl ghrelin.

There was a significant decrease in the average t = +70/150 minute composite appetite VAS rating at fed-saline (4.65 ± 0.22) vs. fasted-saline (7.87 ± 0.30) visits (effect size mean ± SEM −3.22 ± 0.34 [95% CI −2.51, −3.92], t = 9.46, *P* < .0001, paired Student *t* test] (Fig. 2A).

As reported previously [56], there was a significant increase in the average t = +70/150 minute fullness VAS rating at fed-saline (3.64 ± 0.34) vs. fasted-saline (0.78 ± 0.17) visit (effect size 2.86 ± 0.33 [95% CI 2.16, 3.55], t = 8.55, *P* < .0001, paired Student *t* test) (Fig. 2B).

#### Correlations with plasma LEAP2

The postprandial increase in plasma LEAP2 (Δ fed-fasted, average t = +70/150 minutes) positively correlated with the postprandial decrease in appetite VAS rating (Δ fasted-fed, average t = +70/150 minutes) (r_s_ = +0.47, *P* = .026) (Fig. 2C).

However, the postprandial increase in plasma LEAP2 (Δ fed-fasted, average t = +70/150 minutes) did not correlate with the postprandial increase in fullness VAS rating (Δ fed-fasted average, t = +70/150 minutes) (r_s_ = +0.09, *P* = .69) (Fig. 2D).

#### Correlations with plasma AG

The postprandial decrease in plasma AG (Δ fed-fasted, average t = +70/150 minutes) did not correlate significantly with the postprandial decrease in appetite VAS rating (Δ fasted-fed, average t = +70/150 minutes) (r_p_ = +0.16, *P* = .47) (Fig. 2E).

Similarly, the postprandial decrease in plasma AG (Δ fed-fasted, average t = +70/150 minutes) did not correlate significantly with the postprandial increase in fullness VAS rating (Δ fed-fasted, average t = +70/150 minutes) (r_p_ = −0.30, *P* = .18) (Fig. 2F).

#### Correlations with plasma AG/LEAP2 molar ratio

The postprandial decrease in plasma AG/LEAP2 molar ratio (Δ fed-fasted, average t = +70/150 minutes) did not correlate significantly with the postprandial decreases in appetite VAS rating (Δ fasted-fed, average t = +70/150 minutes) (r_p_ = +0.18, *P* = .41) (Fig. 2G).

The postprandial decrease in plasma AG/LEAP2 molar ratio (Δ fed-fasted, average t = +70/150 minutes) showed a trend toward a negative correlation with postprandial increase in fullness VAS rating (Δ fed-fasted, average t = +70/150 minutes) (r_p_ = −0.38, *P* = .082) (Fig. 2H).

### Food Cue Reactivity

#### Correlations with plasma LEAP2

##### High-energy food pictures

**Figure 3.**
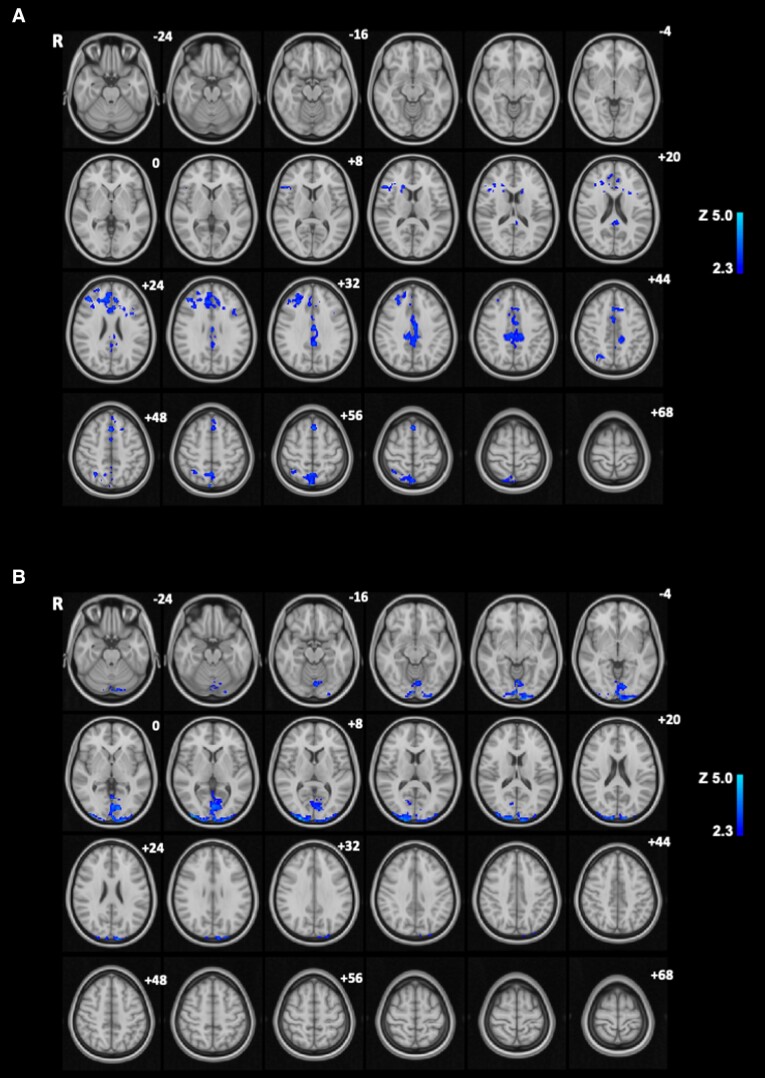

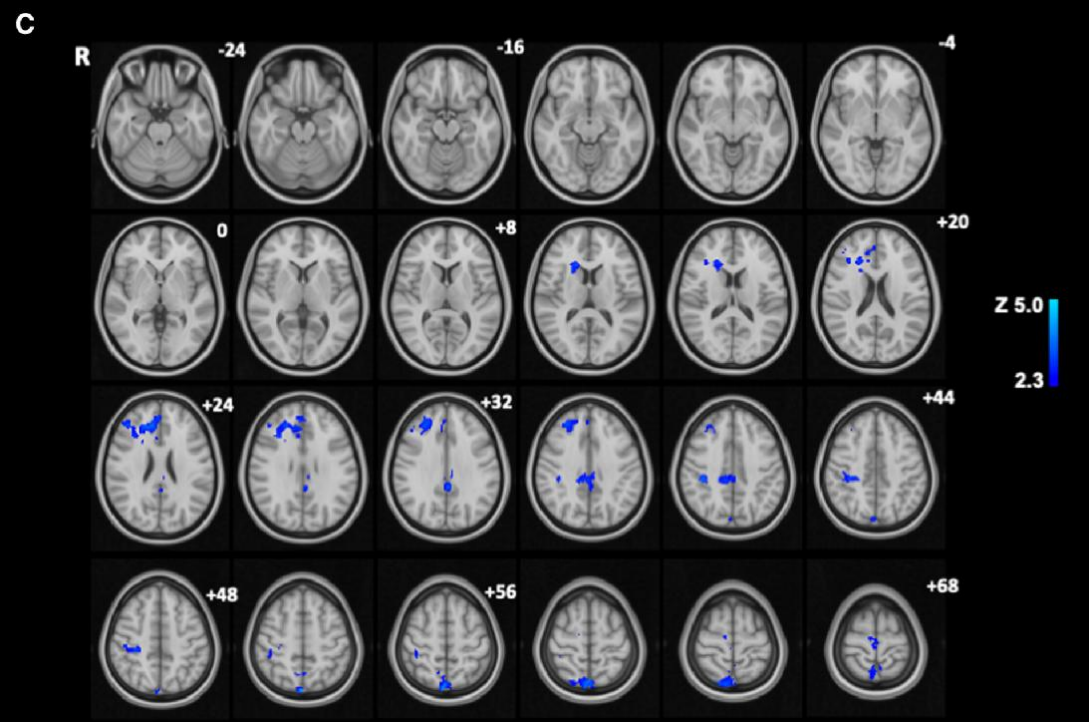
**Whole-brain analysis of correlation between postprandial changes in plasma LEAP2 and BOLD signal during food picture evaluation fMRI task.** Brain regions (blue) showing negative correlations between postprandial changes in plasma LEAP2 (Δ fed-fasted, average of t = +70 and +150 minutes) and postprandial changes (Δ fed-fasted) in BOLD signal during evaluation of pictures of (A) high-energy food vs. objects (B) low-energy foods (vs. objects), and (C) high-energy or low-energy foods (vs. objects) in whole-brain analysis (n = 22, cluster-wise family wise error (FWE) *Z* > 2.3, *P* < .05). Color bar indicates *Z* statistic, *z* coordinates in Montreal Neurological Institute (MNI) space. Abbreviation: R, right.

In whole-brain analysis, there was a significant negative correlation between postprandial changes in BOLD signal to HE food (vs. object) pictures with postprandial changes in plasma LEAP2 in the superior frontal gyrus, paracingulate gyrus, anterior division cingulate gyrus, posterior division cingulate gyrus, precuneus cortex, juxta positional lobule cortex, inferior frontal gyrus pars triangularis, inferior frontal gyrus pars opercularis, frontal pole, middle frontal gyrus, superior division lateral occipital cortex, superior occipital lobule, angular gyrus, and superior parietal lobule (Supplementary Table S1 [25], Fig. 3A).

##### Low-energy food pictures

In whole-brain analysis, there was a significant negative correlation between postprandial changes in BOLD signal to LE food (vs. object) pictures with postprandial changes in plasma LEAP2 in the postcentral gyrus, precentral gyrus, and superior parietal lobule (Supplementary Table S2 [25], Fig. 3B).

##### High-energy or low-energy food pictures

In whole-brain analysis, there was a significant negative correlation between postprandial changes in BOLD signal to HE/LE food (vs. object) pictures with postprandial changes in plasma LEAP2 in the paracingulate gyrus, anterior division cingulate gyrus, middle frontal gyrus, frontal pole, precuneus cortex, superior division lateral occipital cortex, postcentral gyrus, precentral gyrus, anterior division supramarginal gyrus, and posterior division cingulate gyrus (Supplementary Table S3 [25], Fig. 3C).

##### High-energy vs. low-energy food pictures

In whole-brain analysis, there were no significant correlations between postprandial changes in BOLD signal to HE vs. LE food pictures with postprandial changes in plasma LEAP2.

#### Correlations with plasma AG

##### High-energy food pictures

**Figure 4.**
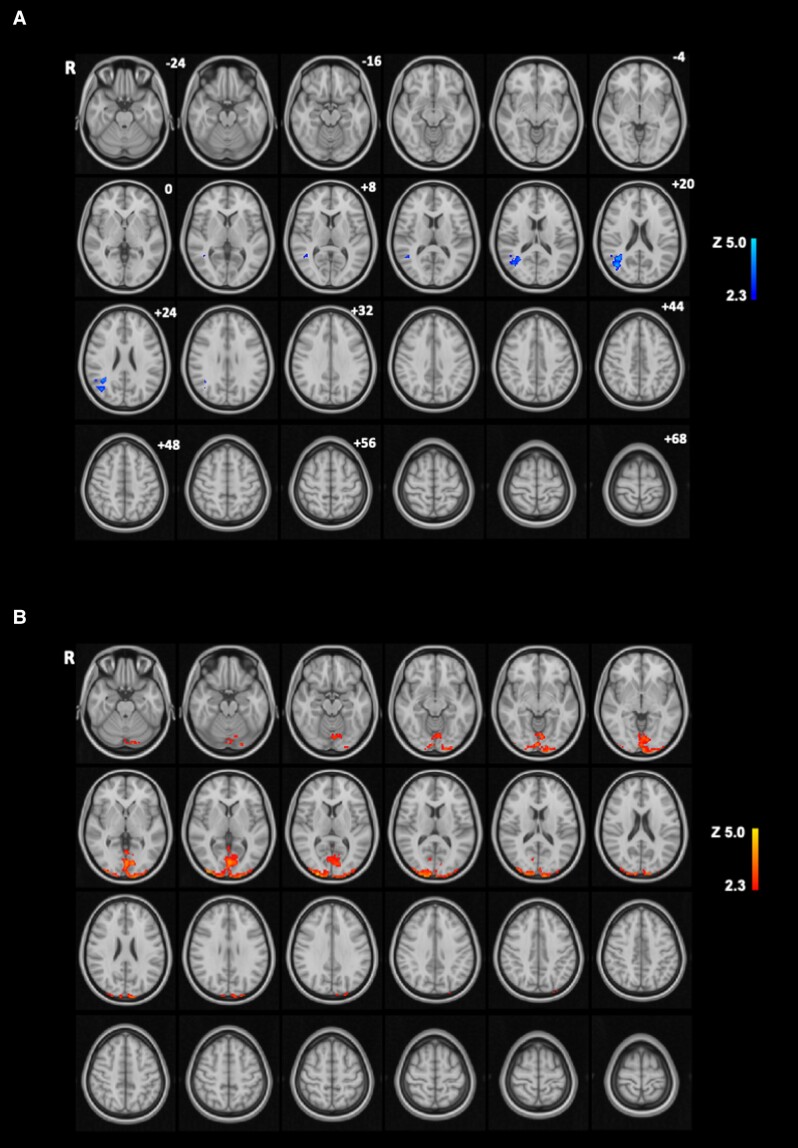
**Whole-brain analysis of correlation between postprandial changes in plasma AG and BOLD signal during food picture evaluation fMRI task.** (A) Brain regions (blue) showing negative correlations between postprandial changes in plasma AG (Δ fed-fasted, average of t = +70 and +150 minutes) and postprandial changes (Δ fed-fasted) in BOLD signal during evaluation of high-energy food pictures (vs. objects), and (B) brain regions (red) showing positive correlations between postprandial changes in plasma AG (Δ fed-fasted, average of t = +70 and +150 minutes) and postprandial changes (Δ fed-fasted) in BOLD signal during evaluation of low-energy food pictures (vs. objects) in whole-brain analysis (n = 22, cluster-wise family wise error [FWE] Z > 2.3, *P* < .05). Color bar indicates *Z* statistic, *z* coordinates in Montreal Neurological Institute (MNI) space. Abbreviations: AG, acyl ghrelin; R, right.

In whole-brain analysis, there was a significant negative correlation between postprandial changes in BOLD signal to HE food (vs. object) pictures with postprandial changes in plasma AG in the angular gyrus, superior division lateral occipital cortex, and temporooccipital part of middle temporal gyrus (Supplementary Table S1 [25], Fig. 4A).

##### Low-energy food pictures

In whole-brain analysis, there was a significant positive correlation between postprandial changes in BOLD signal to LE food (vs. object) pictures with postprandial changes in plasma AG in the occipital pole, intra-calcarine cortex, lingual gyrus, occipital fusiform gyrus, supracalcarine cortex, and inferior division lateral occipital cortex (Supplementary Table S2 [25], Fig. 4B).

##### High-energy or low-energy food pictures

In whole-brain analysis, there were no significant correlations between postprandial changes in BOLD signal to HE/LE food (vs. object) pictures with postprandial changes in plasma AG concentrations (Supplementary Table S3 [25])

##### High-energy vs. low-energy food pictures

In whole-brain analysis, there were no significant correlations between postprandial changes in BOLD signal to HE vs. LE food pictures with postprandial changes in plasma AG.

#### Correlations with plasma AG/LEAP2 molar ratio

In whole-brain analysis, there were no significant correlations between postprandial changes in BOLD signal to HE food (vs. object), LE food (vs. object), HE or LE food (vs. object), or HE vs. LE food pictures with postprandial changes in plasma AG/LEAP2 molar ratios (Supplementary Table S1–S3 [25]).

### Food Appeal Ratings

As previously reported using a different analysis method [56], in repeated measures ANOVA, including visit (fed-saline, fasted-saline) and food energy density (HE food vs. objects, LE food vs. objects) as within-participant factors, there was a trend for a significant energy density × visit interaction on food appeal scores [F(1,21) = 3.57, *P* = .073]. However, there was a significant overall effect of visit (independent of energy density) [F(1,21) = 8.61, *P* = .008] and a significant overall effect of energy density (independent of visit) [F(1,21) = 7.28, *P* = .014] (Supplementary Fig. S4 [25]).

In post hoc analysis, HE food appeal ratings were significantly lower at fed-saline (1.36 ± 0.20) than fasted-saline (1.69 ± 0.20) visit (effect size −0.33 ± 0.06 [95% CI −0.47, −0.18], *P* < .015) (Supplementary Fig. S4 [25]). Food appeal rating was significantly greater for HE food compared with LE food at the fasted-saline visit (effect size 0.41 ± 0.18 [95% CI 0.08, 0.73], *P* = .012), but not at the fed-saline visit (effect size 0.25 ± 0.14 [95% CI −0.14, 0.63], *P* = .31).

In direct comparison, the impact of food intake on food appeal rating tended to be greater for HE food (Δ fed-fasted) (−0.33 ± 0.09) than for LE food (Δ fed-fasted) (−0.17 ± 0.09) (effect size −0.16 ± 0.08 [95% CI −0.018, 0.34], t = 1.86, *P* = .076, paired Student *t* test).

#### Correlations with plasma LEAP2, AG, and AG/LEAP2 molar ratio

There were no significant correlations between postprandial increases in plasma LEAP2 (Δ fed-fasted average t = +70/150 minutes), nor postprandial decreases in plasma AG and plasma AG/LEAP2 molar ratio (Δ fasted-fed average t = +70/150 minutes) with postprandial decreases (Δ fasted-fed) in appeal rating of HE food (vs. objects) (r_s_ = +0.12, *P* = .59; r_p_= +0.06, *P* = .80; r_p_ = +0.20, *P* = .37 respectively) (Supplementary Fig. S5A, C and E [25]).

There were no significant correlations between postprandial increases in plasma LEAP2 nor postprandial decrease in plasma AG with postprandial decreases in appeal ratings of HE vs. LE food (r_s_ = +0.08, *P* = .71 and r_p_ = +0.27, *P* = .22, respectively) (Supplementary Fig. S5B and D [25]). Though there was a trend for a positive correlation between postprandial decreases in plasma AG/LEAP2 molar ratio with postprandial decreases in appeal ratings of HE vs. LE food (r_p_ = +0.40, *P* = .064) (Supplementary Fig. S5F [25]).

### Food Intake at Ad Libitum Meal

**Figure 5.**
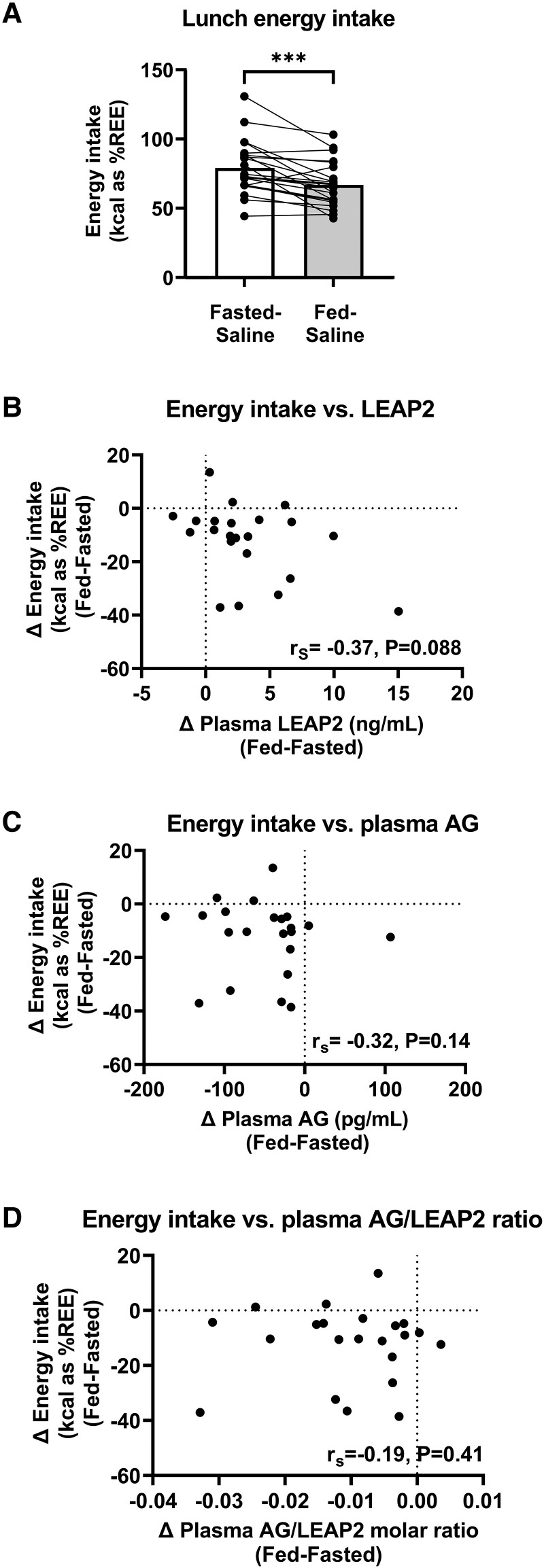
**Energy intake at fed and fasted visits and correlations of postprandial plasma LEAP2, AG, and AG/LEAP2 molar ratio with energy intake.** (A) Energy intake adjusted for kcal as percentage of resting energy expenditure between fed-saline (730 kcal breakfast) and fasted-saline (overnight fast) visits, paired Student *t* test: *P < .05, **P < .01, ***P < .001, ****P < .0001, n = 22. (B-D) Correlation of change in energy intake between fed-saline and fasted-saline visits (Δ fed-fasted) with change in hormones at t = +150 minutes between fed-saline and fasted-saline visits (Δ fed-fasted) for plasma (B) LEAP2, (C) AG, and (D) AG/LEAP2 molar ratio using Spearman (r_S_) correlation coefficients, n = 22. Abbreviations: AG, acyl ghrelin; kcal, kilocalorie; REE, resting energy expenditure.

As previously reported using a different analysis method using unadjusted values [56], there was a significant decrease in REE-adjusted energy intake at fed-saline (67.19 ± 3.50% REE) vs. fasted-saline (79.48 ± 4.11% REE) visits using the Cunningham equation (effect size −12.28 ± 2.94% REE [95% CI −18.39, −6.18], t = −4.18, *P* = .0004, paired Student *t* test] (Fig. 5A). Similar decreases in REE-adjusted energy intake between the fed-saline (70.07 ± 3.66% REE) vs. fasted-saline visits (84.34 ± 4.47% REE) were seen using the Schofield equation (effect size −13.31 ± 3.25% REE [95% CI −20.08, −6.55], t = −4.09, *P* = .0005, paired Student *t* test).

#### Correlations with plasma LEAP2

The postprandial decrease in food intake (Δ fasted-fed kcal as %REE using the Cunningham equation) showed a trend toward a positive correlation with the postprandial increase in plasma LEAP2 (Δ fed-fasted t = +150 minutes) (r_s_ = +0.37, *P* = .088) (Fig. 5B).

A similar correlation coefficient was seen for postprandial increase in plasma LEAP2 (Δ fed-fasted t = +150 minutes) with postprandial decrease in food intake (Δ fed-fasted) expressed as %REE using the Schofield equation (r_s_ = +0.35, *P* = .11).

#### Correlations with plasma AG and AG/LEAP2 molar ratio

The postprandial decrease in food intake (Δ fasted-fed kcal as %REE using the Cunningham equation) did not correlate significantly with the postprandial decrease (Δ fasted-fed t = +150 minutes) in plasma AG (r_s_ = −0.32, *P* = .14) (Fig. 5C) nor plasma AG/LEAP2 molar ratio (r_s_ = −0.19, *P* = .41) (Fig. 5D).

#### Correlations of postprandial changes in gut hormones with BMI

**Figure 6.**
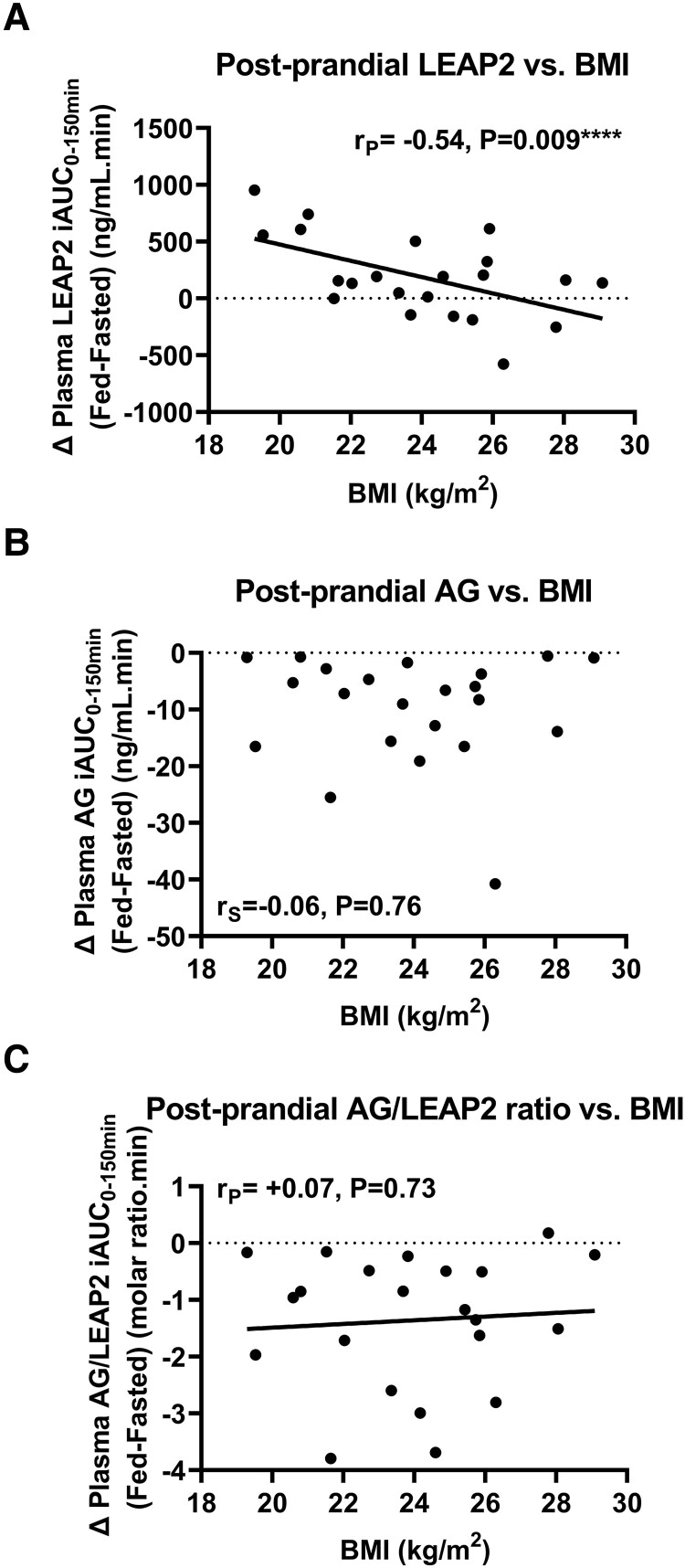
**Correlations of postprandial plasma LEAP2, AG, and AG/LEAP2 molar ratio with BMI.** Correlation of change in incremental value of area under the curve (iAUC) from t = 0 to +150 minutes between fed-saline and fasted-saline visits (Δ fed-fasted) for plasma (A) LEAP2, (B) AG, and (C) AG/LEAP2 molar ratio with BMI using Pearson (r_P_, linear regression line) or Spearman (r_S_) correlation coefficients. Abbreviations: AG, acyl ghrelin; BMI, body mass index.

BMI was negatively correlated with postprandial increase in plasma LEAP2 iAUC_0-150 minutes_ (Δ fed-fasted) (r_p_ = −0.54, *P* = .009) (Fig. 6A). However, BMI was not correlated significantly with postprandial decreases in either plasma AG iAUC_0-150min_ (Δ fasted-fed) (r_s_ = +0.06, *P* = .76), nor plasma AG/LEAP2 molar ratio iAUC_0-150 minutes_ (Δ fasted-fed) (r_p_ = −0.07, *P* = .73) (Fig. 6B, C).

### Correlations of Postprandial Changes in Gut Hormones and Markers of Glucose and Lipid Metabolism

#### Postprandial plasma glucose

As previously reported using a different analysis method [56], there was a significant increase in plasma glucose iAUC_0-150min_ at the fed-saline (20.13 ± 15.35 mmol/L/min) vs. fasted-saline (−13.93 ± 3.52 mmol/L/min) visit (effect size 34.07 ± 14.97 [95% CI 2.93, 65.20], t = 2.28, *P* = .033, paired Student *t*-test) (Supplementary Figure S6A, B [25]).

**Figure 7.**
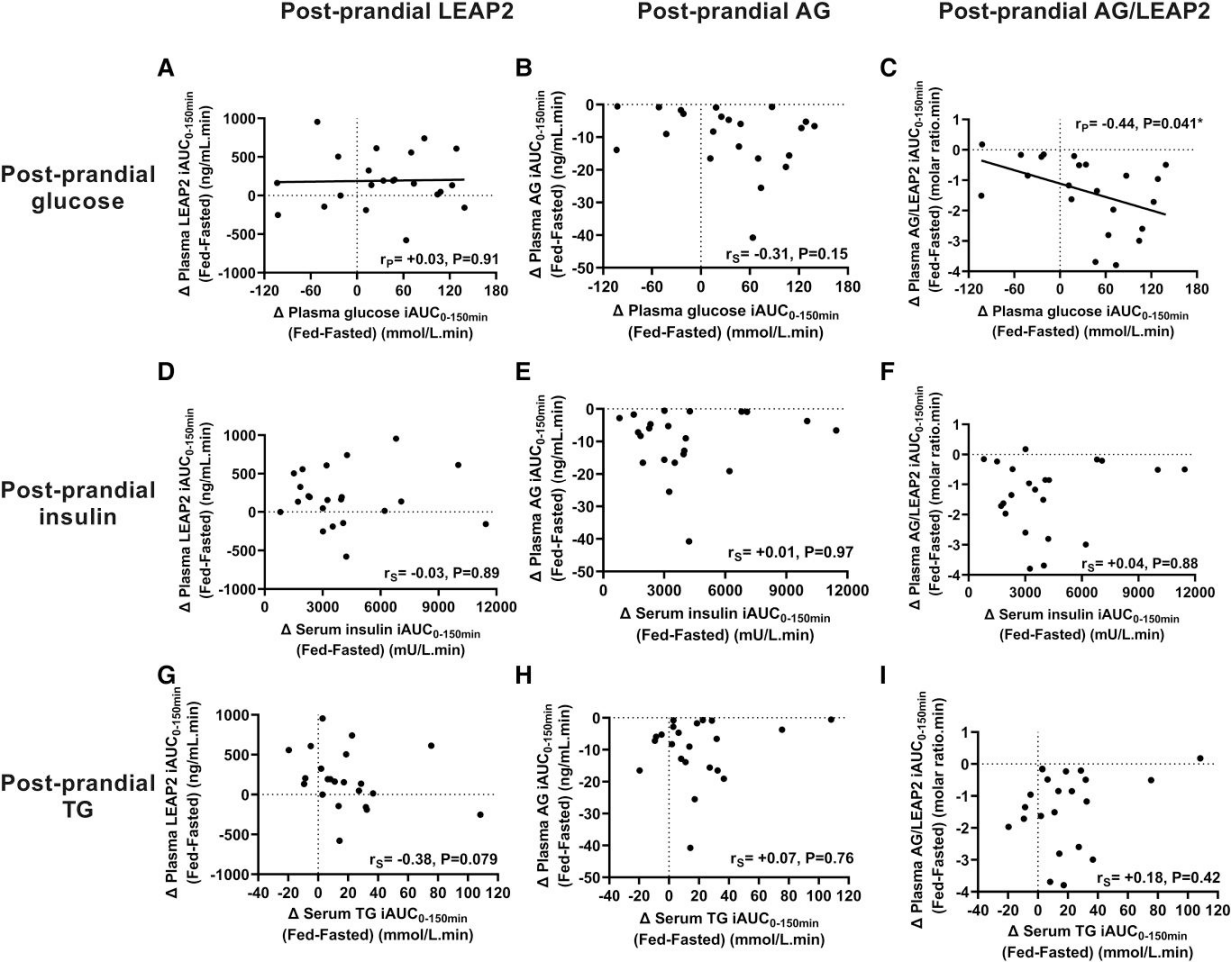
**Correlations of postprandial plasma LEAP2, AG, and AG/LEAP2 molar ratio with postprandial markers of glucose and lipid metabolism.** Correlations of change in incremental value of area under the curve (iAUC) from t = 0, +40, +70, and +150 minutes timepoints (except AG and AG/LEAP2 molar ratio missing t = +40 minute timepoint) between fed-saline and fasted-saline visits (Δ fed-fasted) for (A-C) plasma glucose, (D-F) serum insulin, and (G-I) serum triglycerides with plasma (A, D, G) LEAP2, (B, E, H) AG, and (C, F, I) AG/LEAP2 molar ratio, using Pearson (r_P_, linear regression line) or Spearman (r_S_) correlation coefficients, n = 22. Abbreviations: AG, acyl ghrelin; TG, triglycerides.

The postprandial increase in plasma glucose iAUC_0-150min_ (Δ fed-fasted) was not significantly correlated with the postprandial increase in plasma LEAP2 iAUC_0-150min_ (Δ fed-fasted) (r_p_ = + 0.03, *P* = .91), nor the postprandial decrease in plasma AG iAUC_0-150min_ (Δ fasted-fed) (r_s_ = + 0.31, *P* = .15), but was positively correlated with the postprandial decrease in plasma AG/LEAP2 molar ratio (Δ fasted-fed) (r_p_ = +0.44, *P* = .041) (Fig. 7A–C).

Similar results were seen when excluding the t = +40 minute time point for plasma glucose for the correlations of postprandial increase in plasma glucose iAUC_0-150 minutes_ (Δ fed-fasted) with postprandial increase in plasma LEAP2 iAUC_0-150 minutes_ (Δ fed-fasted) (r_p_ = +0.01, *P* = .93), or the postprandial decrease in plasma AG/LEAP2 molar ratio (Δ fasted-fed) (r_p_ = +0.49, *P* = .022), performed as t = +40 minute time point was missing for plasma LEAP2 values.

#### Postprandial serum insulin

As previously reported using a different analysis method [56], there was a significant increase in serum insulin iAUC_0-150 minutes_ at the fed-saline (median 2,379 mU/L.min [IQR 1,482.5, 3,726.5]) vs. fasted-saline (median 477 mU/L.min [IQR 303, 601]) visit (W-statistic 253, *P* < .0001, Wilcoxon matched-pairs signed-rank test) (Supplementary Fig. S6C and D [25]).

However, there were no significant correlation between the postprandial increase in serum insulin iAUC_0-150 minutes_ (Δ fed-fasted) and postprandial changes in plasma LEAP2, AG, and AG/LEAP2 molar ratio (Δ fed-fasted) (r_s_ = −0.03 to +0.04, *P* = .88-.97) (Fig. 7D-F).

#### Postprandial serum triglycerides

As previously reported using a different analysis method [56], there was a significant increase in serum triglyceride iAUC_0-150 minutes_ at the fed-saline (median 21.93 mmol/L.min [IQR 14.35, 34.16]) vs. fasted-saline (median 10.71 mmol/L.min [IQR 4.21, 16.49]) visit (*Z*-statistic, −3.04; W-statistic, 33; *P* = .002, Wilcoxon matched-pairs signed-rank test) (Supplementary Figure S6E, F [25]).

The postprandial increase in serum triglyceride iAUC_0-150 minutes_ (Δ fed-fasted) showed a trend toward a negative correlation with the postprandial increase in plasma LEAP2 iAUC_0-150 minutes_ (Δ fed-fasted) (r_s_ = −0.38, *P* = .079) (Fig. 7G). However, there was no correlation of the postprandial increase in serum triglycerides with the postprandial decrease in plasma AG iAUC_0-150 minutes_ (Δ fasted-fed) (r_s_ = −0.07, *P* = .76), nor postprandial decrease in plasma AG/LEAP2 molar ratio (Δ fasted-fed) (r_s_ = −0.18, *P* = .42) (Fig. 7H and I).

## Discussion

This human study in adults without obesity demonstrated that increases in plasma LEAP2 after food intake were related to postprandial suppression of eating behavior because: (1) as hypothesized, postprandial increases in plasma LEAP2 positively correlated with the postprandial decrease in appetite ratings, though there was no correlation with the postprandial decrease in plasma AG or AG/LEAP2 ratio; (2) as hypothesized, the postprandial increase plasma LEAP2 tended to positively correlate with the postprandial reduction in energy intake at an *ad libitum* lunch, though there was no trend for plasma AG or AG/LEAP2 ratio; (3) as hypothesized, postprandial increases in plasma LEAP2 positively correlated with postprandial decreases in food cue reactivity (using a food picture evaluation fMRI task) to HE, LE, and HE/LE foods, whereas postprandial decreases in plasma AG positively correlated with the postprandial decrease in food cue reactivity to LE foods. Though contrary to our hypothesis, postprandial decreases in plasma AG negatively correlated with the postprandial decrease in food cue reactivity to HE foods, and there were no correlations of postprandial changes in food cue reactivity with postprandial decreases in plasma AG/LEAP2; (4) contrary to that hypothesized, the postprandial increase in plasma LEAP2 does not appear to be due to the postprandial decreases in plasma AG because these postprandial hormone changes were not positively correlated with each other, and especially as exogenous AG administration did not reduce postprandial plasma LEAP2; (5) contrary to that hypothesized, the postprandial increase in plasma LEAP2 was not positively correlated with postprandial increases in glucose, insulin, or triglycerides, whereas the postprandial decrease in plasma AG/LEAP2 ratio was negatively correlated with the postprandial increase in plasma glucose (mainly driven by the postprandial decrease in plasma AG), though not serum insulin as would be predicted from the literature; and (6) the postprandial increase in plasma LEAP2 was negatively correlated with BMI, in contrast to that previously reported in the literature, with no correlations seen between postprandial decrease in plasma AG or AG/LEAP2 ratio and BMI.

### Postprandial Increase in Plasma LEAP2

The post-prandial increase in plasma LEAP2 was consistent with previous literature [4, 9, 27, 28, 31], interestingly, when examining these multiple human studies, there is a suggestion that the postprandial increase in plasma LEAP2 is proportional to the energy content of meal, though there were some slight differences in macronutrient content of the fixed meals (carbohydrates, 49-55%; fat, 30-35%, protein, 14-18%), nature of meals (4 of 5 meals were liquid meal supplements, but mixed meal in current study), and variation in the timing of postprandial blood sampling between the studies (90, 120, and 150 minutes). However, all studies used the same LEAP2 assay (Phoenix Pharmaceuticals Inc., CA, USA; cat. No. EK-075-40). This influence of meal size on plasma LEAP2 has previously been demonstrated for other appetitive gut hormones such as orexigenic total ghrelin (negative relationship) and anorexigenic peptide YY (positive relationship) [73, 74]. This suggests that plasma LEAP2 may be a further peripheral anorexigenic signal of recent energy intake to the brain to attenuate human eating behavior, especially when integrated with postprandial decreases in plasma AG to decrease the plasma AG/LEAP2 ratio. However, this needs to be examined in future participant studies measuring plasma LEAP2 and AG after consumption of fixed meals of varying caloric value. Furthermore, the influence of intake of different macronutrients on plasma LEAP2 needs to be examined, as previously reported for plasma AG and total ghrelin [2, 75].

In the current study, postprandial plasma LEAP2 increased further from 70 to 150 minutes after food intake, but later time points were unavailable, which should be examined in future studies because peak concentrations might be achieved later. This compares with peak suppression of plasma AG and AG/LEAP2 ratio occuring at 70 minutes in this study. Unfortunately, because of technical problems with between assay variations, we were not able to report exact plasma LEAP2 concentrations at 40 minutes after the meal, but there was no suggestion that plasma LEAP2 peaked at this earlier time point using quality control-adjusted corrected values. This would be consistent with the hepatic (rather than intestinal) source of postprandial circulating LEAP2 suggested from preclinical studies [9, 31].

### Role of GHSR1a-related Hormones in Appetite and Food Intake

A role of endogenous GHSR1a-related hormones in stimulating human appetite and food intake, as opposed to meal preparation and initiation, is disputed. Although exogenous AG simulates appetite ratings and food intake in humans without and with obesity, this is only with markedly supraphysiological plasma AG concentrations [21‐23]. By contrast, low-dose exogenous AG administration to achieve physiological or mildly supraphysiological plasma AG concentrations had no effect on appetite ratings nor food intake in men of normal weight when fed [24]. Chronic administration of an oral GOAT inhibitor (GLWL-01) over 28 days, which markedly decreased plasma AG in adults with obesity and type 2 diabetes mellitus also had no effect on body weight (though this is complicated by plasma AG being low already in obesity) (https://clinicaltrials.gov/ct2/show/results/NCT02377362). Although an oral GHSR1a inverse agonist, PF-5190457, reduced alcohol craving in adults with alcohol use disorder with no effect on glucose metabolism, it has not been examined in normal weight or obesity, and effects on appetite and food intake have not been reported [76‐78].

In the current study, there was a clear postprandial decrease in appetite ratings and *ad libitum* meal energy intake compared with the fasted state. Postprandial increases in plasma LEAP2 positively correlated with postprandial decreases in appetite ratings (though not fullness), with a trend for postprandial decreases in energy intake. However, there were no correlations with postprandial decreases in AG nor AG/LEAP2 molar ratio. These novel findings thus do provide support a role for GHSR1a signaling in stimulating human appetite and food intake, either through antagonism of endogenous AG or inverse agonism at the constitutively active GHSR1a [4, 6, 7]. These results are consistent with the recent finding that administration of exogenous LEAP2 to men without obesity (to achieve mildly supraphysiological plasma LEAP2 concentrations) reduced food intake at an *ad libitum* test lunch, although no decreases in appetite ratings were seen [13].

Other than plasma LEAP2, postprandial increases in circulating metabolites and other hormones, including glucose, triglycerides, insulin, and cholecystokinin, might also contribute to postprandial decreases in appetitive behavior. However, this was not compatible with the time course and duration of postprandial increases in plasma glucose, serum insulin, and triglycerides in this study, whereas at the time of the *ad libitum* test meal, plasma glucose was in fact lower at the fed-saline than fasted-saline visit. Furthermore, there were no significant postprandial increases in anorexigenic peptide YY and glucagon-like peptide-1 hormones in this study [56].

The lack of correlations of postprandial decreases in plasma AG or AG/LEAP2 ratio with postprandial decreases in appetite or food intake in this study suggest that postprandial increases in plasma LEAP2 may be more important than decreases in plasma AG in contributing to postprandial reductions in appetitive behavior. Although this might be related to small sample size (type II error), as discussed previously, a definitive role for endogenous ghrelin (as opposed to GHSR1a signaling) in stimulating appetitive behavior is uncertain. There is great variability in the results of studies correlating endogenous postprandial plasma ghrelin concentrations and measures of eating behavior, including appetite ratings and food intake, likely related to often small samples sizes; differences in study design and obesity status; specificity of ghrelin immunoassays to AG, DAG, or both; use of protease inhibitors to prevent breakdown of AG to DAG; use of absolute or changes in plasma ghrelin; and lack of correction of food intake for body size or resting energy expenditure (see Supplementary References for list of publications [25]). Indeed, the majority of published studies do not show positive correlations between postprandial absolute or decreases in plasma total ghrelin/AG with postprandial absolute or decreases in appetite or hunger ratings, postprandial absolute or increases in fullness, nor postprandial food intake.

### Role of GHSR1a-related Hormones in Food Cue Reactivity

#### Correlations of plasma LEAP2 with food cue reactivity

Interestingly, the current study had the novel finding that there was also a correlation between postprandial increases in plasma LEAP2 and postprandial decreases in food cue reactivity during evaluation of HE food, LE food, and HE vs. LE food pictures in the following brain regions: paracingulate gyrus, anterior and posterior cingulate gyrus, precuneus, juxtapositional lobule cortex, superior and inferior frontal gyrus, frontal pole, middle frontal gyrus, lateral occipital cortex, superior occipital lobule, angular gyrus, and superior parietal lobule.

It is difficult, however, to assign specific tasks to these brain regions in the context of this study, but they include areas involved in reward processing, decision making, and executive function. The relevance of some of these brain regions is highlighted by the findings from several fMRI meta-analyses. A meta-analysis of food cue reactivity found increased BOLD signal to HE food (vs. non-food) stimuli in the following regions that overlapped with the findings of the current study: inferior frontal gyrus and anterior cingulate gyrus [79]. A meta-analysis of brain responses to taste found increased BOLD signal to sweet taste (vs. water or tasteless solution) in these overlapping regions: frontal pole, anterior cingulate gyrus, and inferior frontal gyrus [80]. Furthermore, a meta-analysis of obesity-related differences in food cue reactivity found increased BOLD signal in obesity to passive viewing of food (vs. nonfood) stimuli in the inferior frontal gyrus [81], a region that has shown lower food cue reactivity in those with lower desire to eat [82].

The clinical importance of several of these brain regions is also revealed from post-weight loss reductions of BOLD signal to food stimuli in the precuneus, inferior and middle frontal gyrus, posterior cingulate cortex, and lateral occipital cortex from longitudinal fMRI studies of Roux-en-Y gastric bypass surgery for obesity [83‐86]. Furthermore, reductions in the BOLD signal to HE vs. LE food stimuli after Roux-en-Y gastric bypass surgery have been correlated with reductions in the preference for HE vs. LE food in the precuneus [87], and reductions in the liking of HE vs. LE food in the superior frontal gyrus and anterior cingulate cortex [84].

In addition, a physiological role for some of these brain regions in mediating effects of nutritional state on behavior is supported by findings that several of these brain regions also show decreases in food cue reactivity in the postprandial fed compared with fasted state. In men and children with normal weight, food intake decreased BOLD signal to HE and LE food pictures in the anterior cingulate cortex, superior occipital sulcus, posterior cingulate cortex, and superior frontal gyrus (regions that overlapped with the findings of the current study), and the pregenual cingulate cortex, amygdala, subcallosal gyrus, and orbitofrontal cortex (OFC) [51, 52]. In adults without obesity, food intake decreased BOLD signal to HE vs. LE food pictures in the anterior and posterior cingulate cortex, middle and superior frontal lobe (regions that overlapped with the findings of the current study), and the nucleus accumbens, caudate/putamen, amygdala, anterior insula, OFC, and fusiform gyrus [53‐55].

Therefore, these findings provide novel evidence supporting a role for postprandial increases in endogenous plasma LEAP2 in altering food-cue related brain activity in humans. However, no correlations of postprandial plasma LEAP2 were seen with postprandial changes in food cue reactivity in ventral/dorsal striatum, amygdala, OFC, and hippocampus, regions involved in food reward processing, emotional reactivity, salience, and memory [50, 53, 56, 64, 88]. Future studies will need to examine the effect of exogenous LEAP2 administration on food cue reactivity in humans.

#### Correlations of plasma AG with food cue reactivity

The postprandial decrease in plasma AG positively correlated with postprandial decreases in BOLD signal to LE foods in the occipital pole, intra-calcarine cortex, lingual gyrus, occipital fusiform gyrus, supracalcarine cortex, and inferior division lateral occipital cortex, areas of the visual pathway, a direction that would be hypothesized by a role for AG in stimulating visual attention and processing to food. By contrast, there was an unexplained negative correlation between the postprandial decrease in plasma AG and decrease in food cue reactivity to HE food in the angular gyrus, superior division of lateral occipital cortex, and temporooccipital part of middle temporal gyrus. However, no correlations were seen between postprandial decreases in plasma AG/LEAP2 ratio and changes in food cue reactivity.

Several previous studies have examined correlations of postprandial decreases in endogenous plasma ghrelin with changes in food cue reactivity (pictures or taste) or resting BOLD signal with highly variable results, likely related to differences in study design, participant characteristics including obesity status, sample size, assay of total ghrelin or AG, fMRI analysis methods, and statistical thresholds. However, none of these studies has reported correlations of plasma ghrelin with BOLD signal to food cues in the visual pathways, as seen in the current study [60‐63, 65‐67].

Studies of the effects of exogenous ghrelin administration on brain function using fMRI have also found varied results with a number of different tasks (including passive or active viewing of food pictures, encoding, consolidation or recall of memory to food words, food incentive delay task, Pavlovian conditioning to food odors), with no changes in seen in the occipital lobe in any studies [56, 89‐92]. Changes in resting BOLD signal in the occipital lobe have been seen in one study [90], but not others [93].

### Interrelationship of Postprandial Plasma LEAP2 with Plasma AG

The current study found no evidence for inhibition of plasma LEAP2 by plasma AG to explain their reciprocal fasting and postprandial regulation. Exogenous supraphysiological AG administration did not decrease postprandial plasma LEAP2; furthermore, there was no positive correlation between the postprandial decrease in plasma AG and postprandial increase in plasma LEAP2 (in fact, there was a trend for a negative correlation).

This conclusion is supported by the finding that plasma LEAP2 and liver *Leap2* mRNA expression are unaltered in *Ghsr1a* knockout mice [9]. By contrast, there is a suggestion in rodents that LEAP2 may suppress AG, as in food-restricted mice introduction of adeno-associated virus expressing *Leap2*, increasing plasma LEAP2 to 3 times normal, attenuated the expected rise in plasma AG [4]. However, acute exogenous LEAP2 administration in men without obesity, to increase plasma LEAP2 to ∼2.6 times normal when fed, did not alter plasma AG, though this could be a dose or duration effect rather than species difference [13].

### Relationship of Postprandial Plasma LEAP2 with Glucose and Fat Metabolism

A previous study suggested that postprandial increases in plasma LEAP2 may be regulated by postprandial increases in plasma glucose because of their positive correlation in obesity, though absolute values at multiple time points were correlated in one regression model rather than incremental changes, with no accounting for within-participant factors [27]. However, in the current study of adults without obesity, the lack of positive correlations suggests that postprandial increases in plasma LEAP2 are not driven by postprandial increases in plasma glucose, serum insulin, or triglycerides. Decreases in AG/LEAP2 molar ratio did correlate with postprandial increases in plasma glucose, but this appeared to be driven primarily by postprandial decreases in plasma AG. If glucose is stimulating LEAP2 secretion, it remains unknown if this is via changes in portal vein to liver, arterial glucose to liver or small intestine, or an effect of glucose on the luminal surface of the small intestine whose subsequent absorption into the circulation produces an indirect association.

In a previous study, postprandial increases in plasma LEAP2 positively correlated with BMI across a combined cohort of women with normal weight and obesity, although this did not appear to be present in those without obesity alone, and no adults with overweight were included [27]. However, in the current study of men and women without obesity, higher BMI was associated with lower postprandial increases in plasma LEAP2. This may suggest that there is a U-shaped relationship between postprandial increases in plasma LEAP2 and BMI, though this needs confirmation in larger studies across a range of BMI and both sexes.

### Relationship of Postprandial Plasma AG With Glucose and Fat Metabolism

Ghrelin reduces insulin sensitivity and insulin secretion, and increases GH release and plasma glucose (including via GH action to increase gluconeogenesis) [94]. Hence, as part of a homeostatic feedback loop, insulin is known to reduce plasma AG, apparently via direct actions of insulin on ghrelin-secreting gastric cells, which may also contribute to the postprandial decreases in plasma AG. Insulin perfused into the rat stomach inhibits ghrelin secretion [40, 41]. Furthermore, peripheral insulin administration suppressed plasma AG in wild-type mice but not mice with insulin receptor knockout in ghrelin-expressing cells (GhIR-knockout), while similarly hyperinsulinemic-hypoglycemic and hyperinsulinemic-euglycemic clamps reduced plasma AG in wild-type mice but not in GhIR*-*knockout mice [35]. Furthermore, in 24-hour fasted mice, refeeding reduced plasma AG in wild-type mice but not in GhIR-knockout mice, despite no difference in the postprandial increases in plasma LEAP2 [35]. Similar results were seen in mice with diet-induced obesity, in which in reductions in plasma AG were seen in wild-type mice but not in GhIR-knockout mice, suggesting that hyperinsulinemia is mediating the reduction in plasma AG in obesity [35]. This suggests that postprandial increases in insulin mediates postprandial decreases in AG but not increases in plasma LEAP2 in rodents.

In humans, euglycemic hyperinsulinemic clamps inhibits secretion of total ghrelin in adults without obesity [38, 39], whereas over a 24-hour period plasma, total ghrelin changes reciprocally with serum insulin in adults without obesity [42]. Furthermore, correlational studies implicate a role for postprandial increases in circulating insulin to decrease plasma ghrelin postprandially in humans. In boys with obesity, postprandial insulin AUC negatively correlated with postprandial AUC plasma total ghrelin [44].

However, acute administration of subcutaneous insulin bolus with IV glucose infusion in adults with normal weight did not suppress plasma AG, unlike oral glucose or liquid meal ingestion, suggesting a lack of a role for postprandial insulin secretion in directly suppressing ghrelin secretion [43]. In support of this conclusion, in our study of adults without obesity, the postprandial decrease in plasma AG was not correlated with the postprandial increase in insulin. These differences between studies may be explained by assay of AG rather than total ghrelin and the methodology of correlating postprandial changes in insulin with changes in AG rather than absolute values.

### Limitations

Small samples may also have contributed to type I and type II errors and so confirmation of the study findings with larger samples sizes are needed. Furthermore, because the current study was only correlational, to further examine the influences of LEAP2 on eating behavior in humans, the effects of exogenous LEAP2 administration on appetite and food intake need to be investigated in obesity, and on food cue reactivity in both adults without and with obesity.

Because of the limited duration of blood sampling postprandially, we were not able to determine the timing of the postprandial peak in plasma LEAP2, and so longer duration postprandial or 24-hour studies are required to ascertain the temporal dynamics of LEAP2 secretion. Furthermore, this study did not report plasma LEAP2 concentrations at the earlier timepoint at 40 minutes after food intake, and so an earlier peak of plasma LEAP2 cannot be definitively excluded. This missing timepoint is unlikely to have affected the correlational results with hormonal-metabolic mediators of the postprandial increase in plasma LEAP2 because results excluding the t = +40 minute time point for plasma AG, glucose, serum insulin, and triglycerides gave similar lack of significant correlations.

The sample size was small and sex ratio unbalanced and so it was not possible to examine whether correlations of postprandial plasma LEAP2 with eating behavior differed between sexes. Indeed, sex differences have been seen in body weight and food intake in *Leap2* knockout mice, with greater increases seen in females [47]. The current study did not include adults with obesity that have higher fasting and postprandial plasma LEAP2 [27], and so future examination of relationships between plasma LEAP2 and eating behavior needs to be conducted across all BMI categories, as well as children/adolescents because this study included adults only.

## Conclusions

Taken together, these novel findings are consistent with the hypothesis that postprandial increases in plasma LEAP2 (through its actions to decrease GHSR1a signaling as shown in published preclinical studies) is involved in homeostatic human energy balance by contributing to postprandial reductions in appetite, food intake, and food cue reactivity. Furthermore, these postprandial changes in plasma LEAP2 may be more important than postprandial decreases in plasma AG. The metabolic mechanisms underlying this postprandial increase in plasma LEAP2 remains incompletely understood with no evidence found for a relationship with postprandial changes in AG, glucose, insulin, nor triglycerides.

## Data Availability

Some or all datasets generated during and/or analyzed during the current study are not publicly available but are available from the corresponding author on reasonable request.

## Abbreviations

AG: acyl ghrelin
BIA: bioimpedance analysis
BMI: body mass index
BOLD: blood oxygen level-dependent
DAG: des-acyl ghrelin
fMRI: functional magnetic resonance imaging
GhIR: insulin receptor knockout in ghrelin-expressing cells
GHSR1a: GH secretagogue receptor 1a
GOAT: ghrelin-O-acyl transferase
HE: high-energy
iAUC: incremental area under curve
LE: low-energy
LEAP2: liver-expressed antimicrobial peptide-2
OFC: orbitofrontal cortex
REE: resting energy expenditure
r_p_: Pearson correlation coefficient
r_s_: Spearman correlation coefficient
SEM: standard error of the mean
VAS: visual analog scale
